# Patients with exercise-associated ventricular ectopy present evidence of myocarditis

**DOI:** 10.1186/s12968-015-0204-3

**Published:** 2015-11-21

**Authors:** Michael Jeserich, Bela Merkely, Manfred Olschewski, Simone Kimmel, Gabor Pavlik, Christoph Bode

**Affiliations:** Department for Cardiology and Angiology, Heart Center University of Freiburg, Albert-Ludwigs University Freiburg, Freiburg, Germany; Heart and Vascular Center, Semmelweis University, Városmajor str, 68, 1122 Budapest, Hungary; Department of Medical Biometry and Statistics, University of Freiburg, Freiburg, Germany; Medical Practice, Cardiology and Angiology, Koenigstr. 39, 90402 Nuernberg, Germany; Department of Health Sciences and Sports Medicine, Universitiy of Physical Education, H-1123 Alkotás str. 44, Budapest, Hungary; Koenigstr. 39, 90402 Nuernberg, Germany

**Keywords:** Cardiovascular magnetic resonance, Myocarditis, Pericarditis, Premature ventricular beats, Late gadolinium enhancement, STIR

## Abstract

**Background:**

The origin and clinical relevance of exercise-induced premature ventricular beats (PVBs) in patients without coronary heart disease or cardiomyopathies is unknown. Cardiovascular magnetic resonance enables us to non-invasively assess myocardial scarring and oedema. The purpose of our study was to discover any evidence of myocardial anomalies in patients with exercise-induced ventricular premature beats.

**Methods:**

We examined 162 consecutive patients presenting palpitations and documented exercise-induced premature ventricular beats (PVBs) but no history or evidence of structural heart disease. Results were compared with 70 controls matched for gender and age. ECG-triggered, T2-weighted, fast spin echo triple inversion recovery sequences and late gadolinium enhancement were obtained as well as LV function and dimensions.

**Results:**

Structural anomalies in the myocardium and/or pericardium were present in 85 % of patients with exercise-induced PVBs. We observed a significant difference between patients with PVBs and controls in late gadolinium enhancement, that is 68 % presented subepicardial or midmyocardial lesions upon enhancement, whereas only 9 % of the controls did so (*p* < 0.0001). More patients presented pericardial enhancement (35 %) or pericardial thickening (27 %) compared to controls (21 % and 13 %, *p* < 0.0001). Myocardial oedema was present in 37 % of the patients and in only one control, *p* < 0.0001. Left ventricular ejection fraction did not differ between patients and controls (63.1 ± 7.9 vs. 64.7 ± 7.0, *p* = 0.13).

**Conclusions:**

The majority of patients with exercise-associated premature ventricular beats present evidence of myocardial disease consistent with acute or previous myocarditis or myopericarditis.

## Background

The origin and clinical relevance of exercise-induced premature ventricular beats (PVBs) in patients without structural heart disease such as myocardial infarction, coronary artery disease, left ventricular hypertrophy, cardiomyopathies or significant valvular disease is unknown. From a traditional perspective, PVBs at rest in individuals without structural heart disease are assumed to be benign [1–4]. In contrast, more recent studies [5–8] report that participants with frequent atrial or ventricular premature beats at rest or exercise are at higher cardiovascular risk during follow-up. Some studies report no predictive value in conjunction with the prognostic significance of exercise-associated PVBs [9–11] whereas others detect an association between PVBs during exercise and a higher risk of cardiovascular or all-cause mortality rates [5, 12, 13]. The underlying etiology of premature beats is often unknown. LV function, dimensions, and flow parameters can be measured accurately by cardiovascular magnetic resonance (CMR) [14, 15]. Late gadolinium enhancement (LGE) and oedema sequences (STIR) have been performed in patients with acute or remote myocarditis [13–21].

We hypothesized that patients with exercise-associated premature ventricular beats would present evidence of acute or remote myocarditis or myopericarditis.

## Methods

### Patients

We prospectively studied consecutive patients referred to our department with palpitations and exercise-associated premature ventricular beats. One hundred ninety consecutive patients who were transferred to our magnetic resonance department with confirmed exercise-associated ventricular ectopies were examined between 1.1.2008 and 30.11.2014 (end of recruitment period). The controls were prospectively scanned and recruited if they were free of atrial or ventricular premature beats and had no evidence of structural heart disease as was examined by medical history, echocardiography and exercise test before the magnetic resonance scan.

Inclusion criteria for the patients were documented PVBs during exercise treadmill test with a history of palpitations, fatigue or exertional dyspnoea. Exercise-associated PVBs were defined as one or more ventricular ectopics during exercise including the first minute after exercise. Patients were excluded if they had a history or findings suggestive of or confirmed coronary artery disease (history of myocardial infarction, signs of ischemia on stress tests, transmural scar on echocardiography or subendocardial or transmural scars on magnetic resonance tomography), dilated or hypertrophic cardiomyopathy, congenital heart disease, pulmonary hypertension, LV hypertrophy, significant valvular regurgitation or valvular stenosis, renal failure (creatinine ≥1.8 mg/dl, GFR <30). Further exclusion factors were: chronic alcohol abuse, use of sympathomimetic drugs, Conn syndrome, chronic loop diuretic treatment, chronic use of laxatives or any other causes of hypokalemia.

Informed consent was obtained from each patient and control, and the study protocol conforms to the ethical guidelines of the 1975 Declaration of Helsinki as reflected in a priori approval by the institution’s human research committee. After exclusions, our final cohort comprised 162 patients whom we compared to 70 age- and sex-matched controls without exercise-induced PVBs.

### Cardiovascular magnetic resonance 

All images were acquired on a 1.5 T magnetic resonance system (Intera CV 1.5 T, Philips Medical Systems, Best, the Netherlands) and specifically designed software (Release 11). We used a five-element cardiac phased-array coil combined with a homogeneity correction algorithm (Constant Level AppeaRance; CLEAR) [22]. Constant level appearance is a homogeneity correction applied to compensate for signal inhomogeneity attributable to the surface coils. It is equivalent to a SENSE acquisition with a SENSE factor of one to acquire the sensitivity maps for each synergy coil element (relative to the body coil sensitivity) that can be used to get a perfect uniformity correction [22].

Data acquisition was ECG-triggered. We took 2, 3 and 4-chamber long-axis views and 3-D short-axis volume data assessed by steady-state free precession imaging (field of view 350 mm, matrix 256 × 256, slice thickness 10 mm, no gap, echo time 1.6 ms, repetition time 4.0 ms, flip angle 60°) to evaluate LV function and dimensions. Phase-contrast velocity images in the ascending aorta were obtained to measure stroke volume and rule out significant aortic insufficiency. Functional and morphological data were evaluated using view forum 6.5. (Philips Medical Systems, Best, the Netherlands).

All patients underwent ECG-triggered, T2-weighted, fast spin echo triple inversion recovery sequences (STIR) in a short axis view covering the whole left ventricle (seven to nine acquisitions, slice thickness 10 mm, no gap, field of view 350 mm, matrix size 512 **×** 512, flip angle 90°, echo time 100 ms, TR 2 RR intervals). We measured signal intensity in the myocardial wall and skeletal muscle. We draw five regions of interest into the septum, five into the anterior, five into the lateral, and five into the inferior wall of the myocardium. If the regions were inhomogenous we draw five to 10 regions of interests. This was only the case in 5 % of patients. Five regions of interest were drawn into the skeletal muscles (erector spinae muscle or lattissimus dorsi) with a homogenous signal. Relative myocardial signal intensity was calculated by the ratio of myocardial signal intensity and muscle signal intensity. Resulting from our previous study findings, we set the cut-off value of an elevated ratio of signal intensity in STIR images between myocardial and skeletal muscle was at 2.14 [20, 21, 23].

The homogeneity of the signal intensity of the region of interest has been studied intensively at the start of the pilot study in volunteers and patients. A very good robustness of the performed STIR sequence showing a homogeneous signal at different time-points in the same volunteers was found and a good inter- and intraobserver variability was found [20].

Late gadolinium enhancement (LGE) imaging was obtained in all patients 10 min after the iv. administration of 0.2 mmol/kg gadolinium-diethylenetriaminepentaacetate using 3D inversion recovery turbo gradient echo sequences (2 acquisitions, an inversion time 230 to 280 ms, field of view 330 mm, matrix size 256 × 256, slice thickness 5 mm, no gap, echo time 1,4 ms, TR shortest, flip angle 15°) were optimized for each measurement to guarantee maximum myocardial signal suppression. 3D volume of the left ventricle was obtained covering the complete left ventricle without gap during two breath holds. Pericardial enhancement was defined as local or diffuse contrast enhancement. To exclude partial volume effects, pericardial thickening and enhancement were obtained in short and long axis views. Locally-thickened pericardium was defined as ≥4 mm. All CMR analysis including STIR and LGE measurements were carried out by one experienced CMR specialist who was blinded to the clinical characteristics and history of patients and controls. Clinical data were obtained and collected by our co-workers and study nurse.

### Statistical analysis

Data are presented as means and standard deviations for quantitative variables and as absolute and relative frequencies for categorical variables. Variables between patients and controls were compared using t-tests for quantitative and Chi-square-tests for categorical variables. All tests were two-sided and used a significance level of 0.05 to indicate statistical significance.

## Results

Patient baseline characteristics are shown in Table 1. Nineteen percent of our patients had exertional dyspnoea, 31 % precordial discomfort, 43 % fatigue and weakness, and 61 % palpitations. Mean BNP values were measured in 70 patients and were 67 ± 132 pg/ml, 6 patients had values above 100 pg/ml, one of these patients had a value of 1010 pg/ml. Troponin values were elevated in 3 patients. Control subjects did not differ regarding age and sex. Functional parameters are listed in Table 2. Patients and controls had normal LV-function and dimensions. The patients’ LV ejection fraction was not significantly different from those of the controls. Cardiac output was modestly higher in controls compared to patients (Table 2). STIR measurements were unavailable in six patients and five controls. Mean VES frequency during exercise including the first minute after exercise was 29.1 ± 69.1. Forty-one patients had one or more couplets, 15 triplets or ventricular runs.

**Table 1:** Clinical characteristics of our 162 patients

Variable	Patients
Mean age, years (SD)	57.4 (12.8)
Female (%)	54 (33)
Height, cm (SD)	174.8 (8.4)
Weight, kg (SD)	80.1 (13.9)
Systolic blood pressure (mm Hg, SD)	139.2 (18.8.)
Diastolic blood pressure (mm Hg, SD)	82.5 (10.1)
Potassium (mEq/L)	4.5 (0.6)
Palpitations (%)	98 (60.5 %)
Weakness, fatigue (%)	69 (42 %)
Dyspnea (%)	30 (18.5 %)
Precordial pain	50 (31 %)
Additional premature ventricular beats at rest	67 (41 %)

Standard deviation or percentage in parentheses

**Table 2:** Magnetic resonance functional measurements in patients and controls

Variable	Patients (*n* = 162)	Controls (*n* = 70)	P value
Heart rate, beats per minute	76.6 ± 16.7	71.8 ± 13.4	0.11
LV ejection fraction, %	63.1 ± 8.0	64.7 ± 7.0	0.13
LV stroke volume, ml	97 ± 17	97 ± 21	0.86
LV end-diastolic volume, ml	155 ± 34	153 ± 36	0.69
LV end-diastolic diameter, mm	51.1 ± 5	52.3 ± 4	0.08
Cardiac output, L/min	6.5 ± 1.5	7.0 ± 1.6	0.03
STIR of the left ventricular myocardium	590 ± 112	536 ± 115	0.007
STIR of the skeletal muscle	294 ± 60	299 ± 54	0.64
STIR myocardium/skeletal muscle	2.0 ± 0.3	1.8 ± 0.3	0.001
Contrast enhancement, %	67.9	8.5	<0.0001

LV indicates left ventricular. Values are expressed as mean ± standard deviation

STIR: T2-weighted, fast spin echo triple inversion recovery sequences. Values are expressed as mean ± standard deviation

### Oedema

**Fig. 1:**
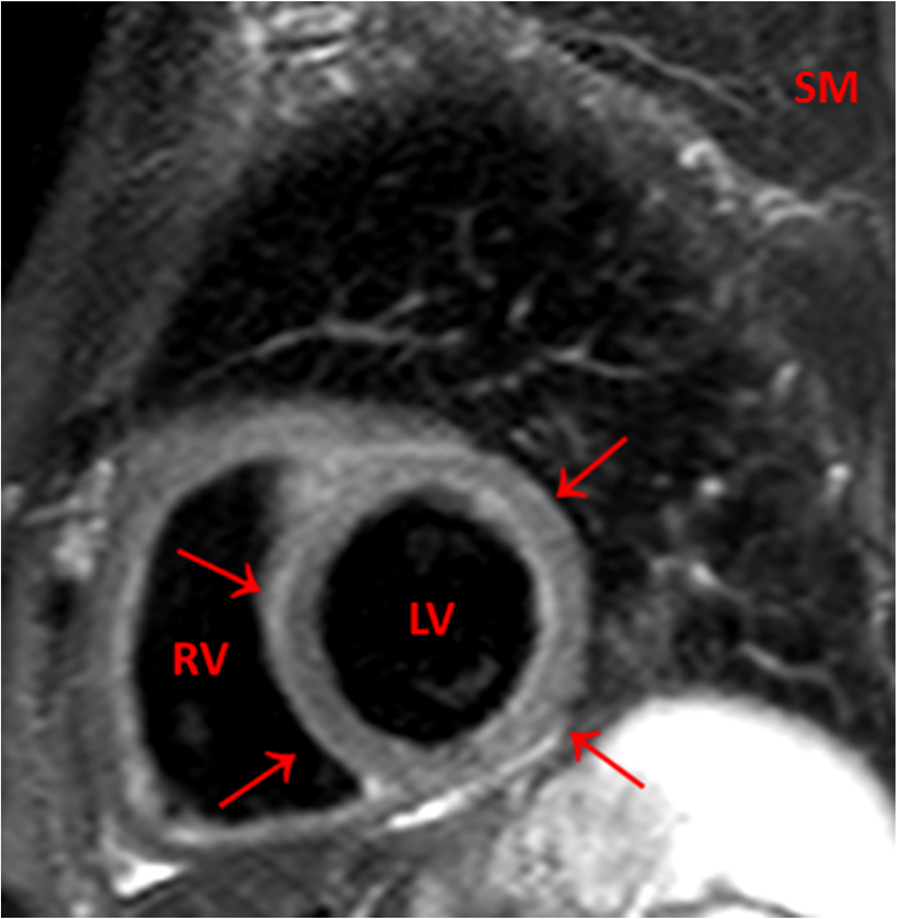
An exemplary T2-weighted (STIR) image of a 57 year old female patient with a viral respiratory viral infection 3 weeks ago and exercise induced PVBs. Globally enhanced signal intensity of the myocardium of the left ventricle (*arrows*) compared to skeletal muscle. The ratio of signal intensity between myocardial and skeletal muscle was elevated (2.26). LV: Left ventricle. RV: Right ventricle. SM: Skeletal muscle

Sixty out of 162 (37 %) patients displayed elevated T2-weighted CMR (STIR) values indicating oedema (ratio of signal intensity in STIR images between myocardial and skeletal muscle ≥ 2.14) and 102 patients had normal values. Figure 1 depicts one patient’s STIR image.

There was no significant difference in either LV-function or LV-volumes (EF 62.6 ± 7.9 % vs. 63.4 ± 7.9 %, *p* = 0.53, left ventricular enddiastolic volume 153 ± 35 ml vs. 156 ± 33 ml, *p* = 0.62) between patients with and without oedema. One control revealed a modestly-elevated STIR ratio but no symptoms.

### Contrast enhancement

**Fig. 2:**
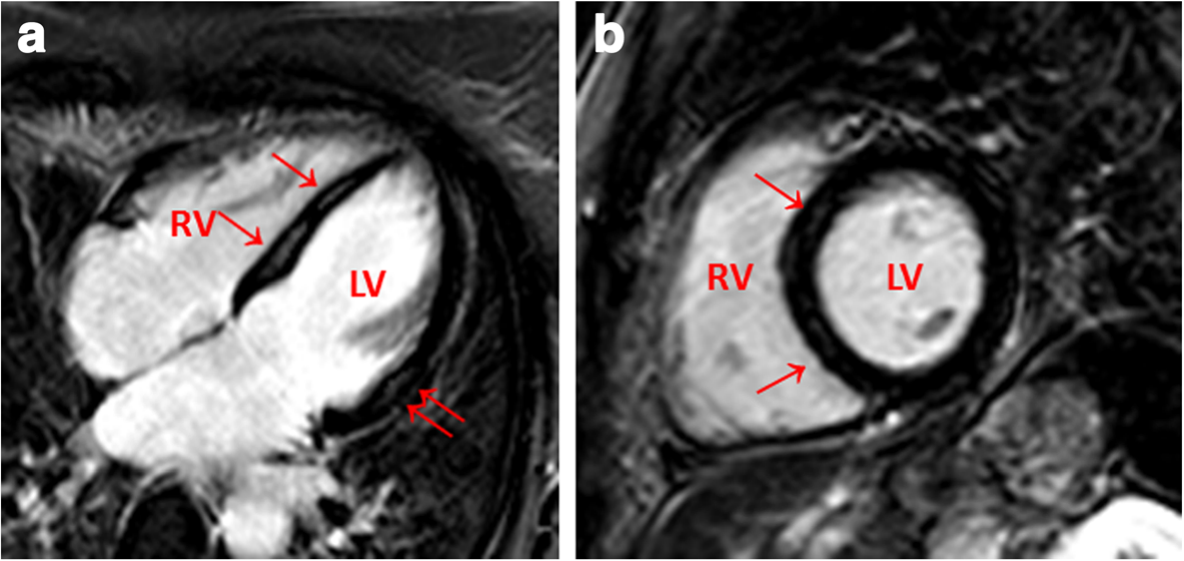
**a**/**b** Late-enhancement image of one patient with exercise induced PVBs. Note the patchy enhancement of the midwall septal (↑) and lateral wall (↑↑). Four and two-chamber view. LV: Left ventricle. RV: Right ventricle

**Fig. 3:**
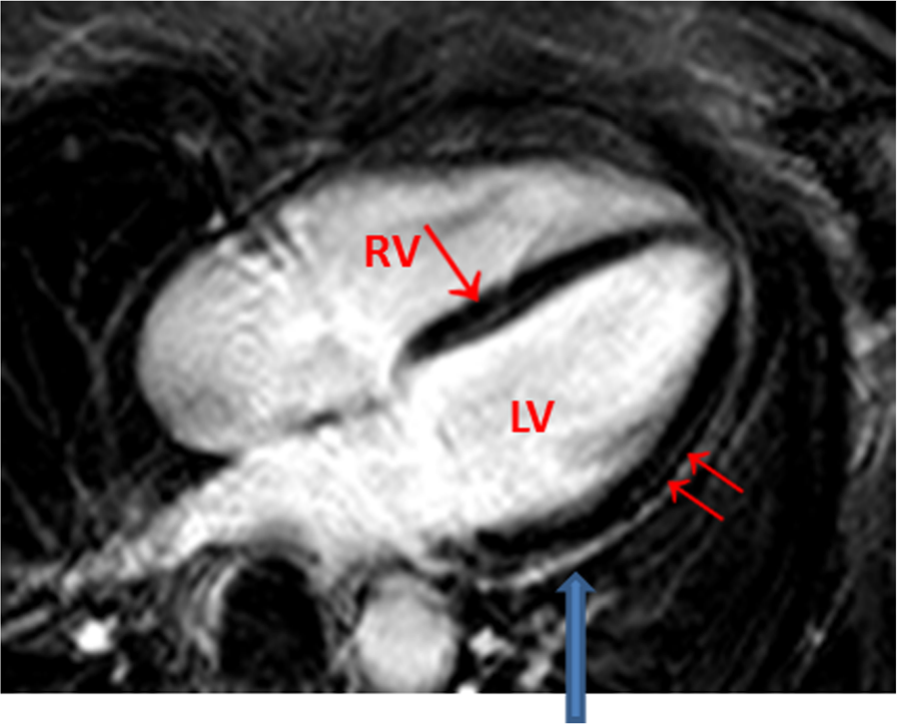
Late-enhancement image of a 59 year old female with long lasting respiratory tract infect, palpitations and exercise induced PVBs. Enhancement of the midwall septal (↑) and lateral wall (↑↑). In addition, lateral pericardial enhancement is visible (↓) Four-chamber view. LV: Left ventricle. RV: Right ventricle

Contrast enhancement was present in the myocardium of 110/162 patients and only in 6/70 controls (*p* < 0.0001, Table 2). Regions of contrast enhancement were usually patchily distributed and originated primarily from the epicardial quartile or midmyocardial wall location, with one or several foci within the myocardium usually located in the septum and lateral free wall, less often in the inferior or anterior areas. Figure 2a/b depict an example of one patient. The location was septal in 45 %, lateral in 27 %, septal and lateral in 9 % of all patients. Figure 3 presents the late-enhancement image of a 59-year-old female with a long-lasting respiratory tract infection and septal and lateral foci. Five patients presented inferior and one patient apical distribution. Thirty-five percent of all patients displayed mild-to-moderate pericardial contrast enhancement, usually distributed locally in the lateral or anterolateral part of the pericardium, compared to 21 % of the controls, *p* < 0.001. Twenty-seven percent of the patients presented locally-thickened pericardium (≥4 mm) primarily in the lateral or anterolateral parts of the pericardium, compared to 13 % of the controls, *p* < 0.001. Minor, not hemodynamically-relevant pericardial effusions were present in 18 % of patients and in 3 controls. The controls’ effusions were small enough as to be invisible on echocardiography.

Twenty-three patients revealed no myocardial or pericardial anomalies, whereas we detected myocardial or pericardial anomalies in 85 % of patients with exercise-induced PVBs.

## Discussion

The present study provides evidence of myocardial anomalies in the vast majority of patients with exercise-associated premature beats. Eighty-five percent of our patients presented signs of acute or remote myocarditis or myopericardits despite having normal LV function. About two-thirds of our patients presented a subepicardial or/midmyocardial late contrast-enhancement typical of myocarditis [18, 19, 24, 25]. In addition, about a third of patients displayed hints of pericardial involvement such as pericardial enhancement, local thickening or small pericardial effusions. Moreover, about a third of our patients presented myocardial oedema. This could have important clinical consequences, since patients with exercise-induced ventricular arrhythmias and myocardial oedema can be at risk for sudden cardiac death during exercise [26].

Some studies of the prognostic significance of ventricular arrhythmia during exercise have assumed that PVBs are benign [9–11], whereas others detected an association between PVBs and greater risk of cardiovascular or all-cause mortality rates [5, 12, 13]. There is recent evidence that frequent PVBs may be involved in the pathogenesis of heart failure in patients with LV dysfunction [27, 28] and that some patients with initially-normal LV function developed a reduction in ejection fraction during an observation period lasting five to six-years [2]. In our study, patients and controls displayed normal LV function but it is well known that some patients with myocarditis develop dilated cardiomyopathy over the following years [29, 30] that may explain their elevated future risk.

Sudden cardiac death in athletes [31] and in the general population is not infrequently due to undiagnosed myocarditis [32, 33]. It has long been known that increased sympathetic nerve activity during physiologic stress (exercise, swimming, emotion, arousal, loud noise, etc.) has profound influence on the heart’s electrical and contractile functions. In the predisposed heart, these stressors may lead to ventricular tachyarrhythmias and sudden death [34]. The extrasystoles observed in our study during exercise testing may also occur during daily activities involving exertion, exercise, or during athletic activities. It is hypothesized that the higher risk of cardiovascular or all-cause mortality in patients with PVBs observed in some studies [5, 12, 13] might be due in part to higher rates of sudden cardiac death. It is thus important to reveal any underlying etiology that might predispose patients to risk. The myocardial oedema observed in 37 % of our patients may be just such a predisposing factor. CMR is considered an important diagnostic tool for myocarditis [18], and an increased signal in T2-weighted images is a typical finding in patients with acute myocarditis [17, 20, 23, 35, 36] Usually, oedema is located in the ventricular wall’s epicardial layer during the acute phase of focal myocarditis. It is reasonable to assume that ventricular ectopic beats may be generated in these areas.

LGE has been investigated in controlled studies with patients suspected of having myocarditis [17, 35, 37]. It revealed a typical subepicardial or midmyocardial late contrast-enhancement pattern. In our study, regions of contrast enhancement showed a pattern typical of non-ischemic injury with subepicardial or midmyocardial distribution that ranged from one to several foci, and from almost normal to diffusely-enhanced myocardium. The predominant areas were the septum and lateral free wall, consistent with other reports [35, 38]. We observed no contrast enhancement involving the subendocardial layers as would be typical for myocardial infarction, supporting the presumption of a chronic degenerative or inflammatory process as a cause of the contrast enhancement.

Pericardial effusion has been reported in 32 to 57 % of patients with myocarditis [39–41]. A quarter of our patient cohort had very small pericardial effusion, supporting the notion of pericarditis accompanying myocarditis. This is further strengthened by our having detected mild to moderate contrast enhancement of the pericardium and locally-thickened pericardium in about 30 % of our patients, findings typical of pericarditis [41, 42].

The aforementioned working groups [5, 9–13] did not address the origin of the PVBs. We observed oedema and/or scar as a pathological substrate in most of our patients. Other potential sources of PVBs such as hypertrophy, renal insufficiency, valvular heart disease, or cardiomyopathies were also ruled out by our exclusion criteria. Our patients presented no clinical evidence of systemic disorders like sarcoidosis or muscular dystrophy in which LGE may also be present [43, 44]. We observed no evidence of long-QT syndrome, catecholaminergic polymorphic ventricular tachycardia, early repolarisation or brugada syndromes which would have revealed prognostic information for the patient [45]. 4 Thus, our study provides evidence that exercise-associated ventricular arrhythmias may be caused by acute or previous inflammatory tissue injury or underlying chronic structural heart disease.

## Conclusions

We conclude that the majority of patients with exercise-associated premature ventricular beats present evidence of myocardial disease consistent with acute or previous myocarditis or myopericarditis.

## Abbreviations

CMR: Cardiovascular magnetic resonance
ECG: Electrocardiogram
EF: Ejection fraction
PVBs: Premature ventricular beats
LV: Left ventricular, left ventricle
LGE: Late gadolinium enhancement
STIR: Fast spin echo triple inversion recovery sequence (s)
